# Armed and Ready: Transcriptional Regulation of Tissue-Resident Memory CD8 T Cells

**DOI:** 10.3389/fimmu.2018.01770

**Published:** 2018-07-30

**Authors:** Felix M. Behr, Ammarina Chuwonpad, Regina Stark, Klaas P. J. M. van Gisbergen

**Affiliations:** ^1^Department of Hematopoiesis, Sanquin Research and Landsteiner Laboratory AMC/UvA, Amsterdam, Netherlands; ^2^Department of Experimental Immunology, Academic Medical Center, Amsterdam, Netherlands

**Keywords:** T cell diferentiation, tissue-resident memory T cells, transcription factors, homolog of Blimp-1 in T cells, BLIMP-1, Notch, RUNX3, secondary responses

## Abstract

A fundamental benefit of immunological memory is the ability to respond in an enhanced manner upon secondary encounter with the same pathogen. Tissue-resident memory CD8 T (T_RM_) cells contribute to improved protection against reinfection through the generation of immediate effector responses at the site of pathogen entry. Key to the potential of T_RM_ cells to develop rapid recall responses is their location within the epithelia of the skin, lungs, and intestines at prime entry sites of pathogens. T_RM_ cells are among the first immune cells to respond to pathogens that have been previously encountered in an antigen-specific manner. Upon recognition of invading pathogens, T_RM_ cells release IFN-γ and other pro-inflammatory cytokines and chemokines. These effector molecules activate the surrounding epithelial tissue and recruit other immune cells including natural killer (NK) cells, B cells, and circulating memory CD8 T cells to the site of infection. The repertoire of T_RM_ effector functions also includes the direct lysis of infected cells through the release of cytotoxic molecules such as perforin and granzymes. The mechanisms enabling T_RM_ cells to respond in such a rapid manner are gradually being uncovered. In this review, we will address the signals that instruct T_RM_ generation and maintenance as well as the underlying transcriptional network that keeps T_RM_ cells in a deployment-ready modus. Furthermore, we will discuss how T_RM_ cells respond to reinfection of the tissue and how transcription factors may control immediate and proliferative T_RM_ responses.

## Introduction

CD8 T cell responses are an essential component of the adaptive immune system that serves to achieve sterile clearance after infection with intracellular pathogens as well as long-term protection against reinfection. To enable protective CD8 T cell responses against a wide spectrum of microbial threats, an extensive repertoire of naïve CD8 T cells is maintained. The diversity within the T cell repertoire is so large that, despite the millions of naïve CD8 T cells, each T cell specificity is only represented by a population in the order of 100–1,000 cells in mice (1–3). Strikingly, these few precursor cells are able to mount robust T cell responses that eliminate virally infected cells to completion within about 1–2 weeks. The efficiency of CD8 T cell responses depends on the highly effective recruitment of naïve CD8 T cells (4), their rapid proliferation resulting in a more than 1,000-fold expansion in about a week (5), and in the acquisition of effector functions by the differentiation into effector CD8 T cells (6). Important effector functions of CD8 T cells include the production of the pro-inflammatory cytokine IFN-γ and the cytotoxic mediators perforin and granzyme B. These effector molecules assist in the activation and recruitment of other immune cells as well as in the elimination of infected cells, respectively. After resolution of infection, most of the effector CD8 T cells undergo apoptosis, resulting in contraction of the CD8 T cell response into an about 10-fold reduced population of memory cells (7, 8) that can be maintained for decades in men. Specific memory CD8 T cells are maintained at a higher frequency than naïve CD8 T cells, which enables them to establish secondary CD8 T cell responses with faster kinetics and of larger magnitude. In this manner, memory CD8 T cells can provide up to life-long protection against re-encounter with the same pathogen (6). Memory CD8 T cells do not only have a numerical advantage, they also display superior qualitative characteristics to provide improved protective immunity compared to naïve T cells (9).

### Subsets of Memory CD8 T Cells

Distinct subsets of memory CD8 T cells have been recognized that contribute to enhanced recall responses in different ways and at separate sites (10). Central memory CD8 T (T_CM_) cells express lymph node (LN) homing molecules such as the CC-chemokine receptor 7 (CCR7) and adhesion molecules such as L-selectin (CD62L) that provide access to secondary lymphoid organs. Due to these properties, T_CM_ cells retain the capacity of naïve CD8 T cells to survey the secondary lymphoid organs for cognate antigens. In contrast, effector memory CD8 T (T_EM_) cells express low levels of CCR7 and CD62L and gain access to the non-lymphoid tissues (11), which enables these memory CD8 T cells to directly patrol the peripheral tissues for immune surveillance. T_CM_ and T_EM_ cells continually recirculate through blood and lymph to survey LN and peripheral tissues, respectively. Recent evidence suggests further heterogeneity within the circulating memory CD8 T cell pool, where expression of the fractalkine receptor CX3CR1 identifies three subsets with distinct migratory properties (12). These include CX3CR1^low^ T_CM_ cells, CX3CR1^int^ peripheral memory T (T_PM_) cells, which survey peripheral tissues, and CX3CR1^high^ T_EM_ cells, which are largely confined to the vasculature (12). Upon recognition of reinfection, T_CM_, T_PM_, and T_EM_ cells mount secondary responses, which involve proliferation and differentiation into secondary effector cells to target the re-invading pathogen.

Next to T_CM_, T_PM_, and T_EM_ cells, a fourth subset of memory CD8 T cells, tissue-resident memory CD8 T (T_RM_) cells, has been identified. In contrast to the circulating memory populations, T_RM_ cells permanently reside within the peripheral tissues after infection without accessing the blood or the lymph (13, 14). The non-recirculating nature of T_RM_ cells has been experimentally demonstrated in different ways. Intravascular antibody injection does not label T_RM_ cells within skin, lungs, and small intestine in contrast to circulating memory CD8 T cells within the bloodstream (15, 16). However, intravascular labeling cannot distinguish circulating memory CD8 T cells transiently passing through the tissues from T_RM_ cells that permanently reside in these tissues. Another exception in this context are liver T_RM_ cells, which reside on the inside of the liver sinusoids in direct contact with the blood (17, 18). Further experiments employing parabiosis, in which the bloodstream of two mice is conjoined, demonstrated that, while circulating memory CD8 T cells rapidly establish equilibrium, T_RM_ cells are permanently retained in peripheral tissues within their host (14, 19–21). The inability of T_RM_ cells to exit donor tissue upon engraftment into recipients has also provided experimental evidence of tissue residency of memory CD8 T cells (13). Quantitative microscopy has shown that T_RM_ cells are more prevalent than circulating memory cells in the non-lymphoid tissues, suggesting that T_RM_ cells form a substantial fraction of the memory repertoire (21). T_RM_ cells do not contribute to systemic immune surveillance, but they establish residence at strategic locations, such as sites, where the primary infection has occurred, positioning them at the frontline of the antimicrobial defense. In this manner, T_RM_ cells are able to mediate border patrol for improved protection against reinfection within the peripheral tissues.

### Phenotype of T_RM_ Cells

**Figure 1:**
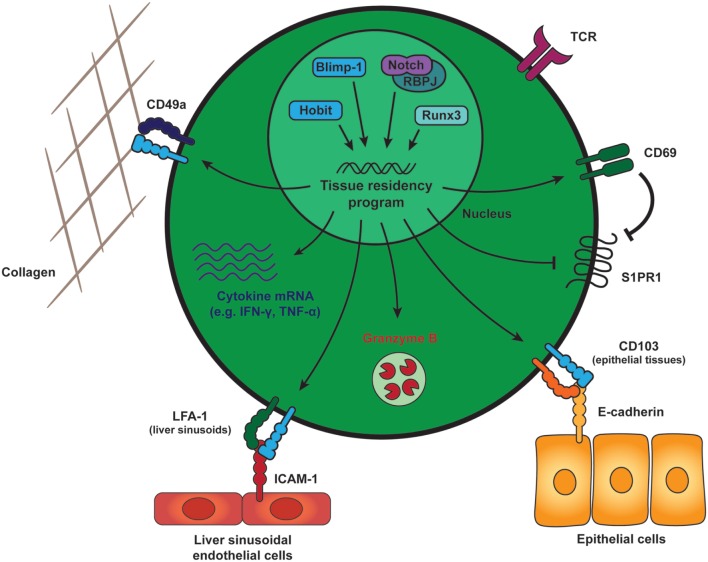
General features of tissue-resident memory CD8 T (T_RM_) cells. Generation and maintenance of T_RM_ cells is regulated by a distinct set of transcription factors, including Runx3, Blimp-1, and its homolog of Blimp-1 in T cells as well as the transcriptional activator Notch. These transcription factors instruct a tissue-residency program that allows for the long-term retention and maintenance of T_RM_ cells within peripheral tissues. T_RM_ cells across tissues maintain expression of CD69, which promotes tissue residency by interfering with spingosine-1 phosphate receptor (S1PR1) function. S1PR1 mediates egress of T cells into the circulation and its downregulation is a core characteristic of T_RM_ cells. In many tissues, T_RM_ cells also express high levels of CD49a, an adhesion molecule binding to collagen (in complex with β1 integrin) to establish tissue residency. The α_E_ integrin CD103 is expressed by mucosal T_RM_ cells and may contribute to tissue retention by interaction with E-cadherin on the surrounding epithelial cells. In liver sinusoids, local T_RM_ cells upregulate LFA-1, which supports their tissue residence by binding to ICAM-1 on liver sinusoidal endothelial cells. In addition to these adhesion molecules, T_RM_ cells in many tissues are characterized by elevated transcript levels encoding for pro-inflammatory cytokines, e.g., IFN-γ and TNF-α, and protein expression of the cytotoxic serine protease granzyme B. Abbreviations: Runx3, Runt-related transcription factor 3; Blimp-1, B lymphocyte-induced maturation protein-1; Hobit, homolog of Blimp-1 in T cells; RBPJ, recombining binding protein suppressor of hairless; LFA-1, lymphocyte function-associated antigen-1; ICAM-1, intercellular adhesion molecule 1; IFN-γ, interferon γ; TNF-α, tumor necrosis factor α.

Tissue-resident memory CD8 T cells can be distinguished from their circulating counterparts through the expression of key cell surface molecules that include CD69 and the α_E_ integrin, CD103 (Figure 1). CD69 is ubiquitously expressed early after activation on T cells, but exclusively T_RM_ cells are able to constitutively maintain CD69 expression under steady state conditions. The majority of T_RM_ cells throughout different tissues express CD69, but parabiosis studies have demonstrated the existence of T_RM_ populations that lack CD69 expression (21, 22). CD69 contributes to the establishment of tissue residency by interfering with spingosine-1 phosphate receptor (S1PR1) function (23, 24). To maintain residency, T_RM_ cells limit expression of tissue exit receptors such as the S1PR1 (25, 26). S1PR1 responds to its ligand S1P that is released by endothelial cells in blood and lymph to attract circulating memory T cells from the tissues into the circulation. In T_RM_ cells, CD69 mediates the internalization and degradation of S1PR1, which results in removal of S1PR1 from the surface and limits the migratory capacities of these memory cells (Figure 1). T_RM_ cells do not form upon forced expression of S1PR1, demonstrating the incompatibility of this pathway with establishment of tissue residency in memory CD8 T cells (26). Expression of CD103 appears to be enriched in T_RM_ cells within mucosal compartments, including the skin, lungs, reproductive tract, salivary glands, and small intestine (25, 27–29). A large fraction of CD103^+^ T_RM_ cells within these tissues locates near or within the epithelium. Epithelial cells express the adhesion molecule E-cadherin, and interaction between CD103 (as part of the αEβ7 integrin) and E-cadherin has been shown to mediate the adhesion between T lymphocytes and epithelial cells (30, 31), suggesting an important role in the retention of T_RM_ cells within epithelial tissues (Figure 1). T_RM_ cells are present outside of the epithelia within a wide array of tissues, including the lamina propria of the small intestine, parenchyma of internal organs, such as the brain, kidney, liver, and within the secondary lymphoid organs (32–34). T_RM_ cells within these tissues largely lack expression of CD103 and may employ other adhesion molecules for retention within the tissues. For instance, T_RM_ cells within the liver express lymphocyte function-associated antigen-1 (LFA-1), which is essential for these cells to mediate interactions with intercellular adhesion molecules on liver sinusoidal endothelial cells (18) (Figure 1). Many T_RM_ cells throughout tissues also express high levels of CD49a, which, in complex with β1 integrin, binds collagen within the extracellular matrix to establish tissue residency (35) (Figure 1). Therefore, elevated expression of adhesion molecules, such as CD103, LFA-1, and CD49a characterizes populations of T_RM_ cells and distinguishes them from circulating memory CD8 T cells.

The identification of human T_RM_ cells largely relies on phenotypic markers, due to difficulties in experimentally addressing the migratory behavior of human memory T cells *in vivo*. Considerable numbers of T_RM_-type memory CD8 T cells co-expressing CD69 and CD103 have been found within human tissues, including skin, lung, liver, and intestines (33, 36–38), suggesting that humans also contain a resident compartment of memory CD8 T cells. These human T_RM_ cells share characteristics with their murine counterparts (33, 39, 40), as determined by transcriptional and phenotypic profiling. Similar to the transcriptional profile of murine T_RM_ cells, the core signature of human T_RM_ cells includes upregulated genes associated with the establishment of tissue residency such as CD49a and downregulated genes associated with tissue egress, e.g., S1PR1 and CCR7 (40).

Tissue-resident memory CD8 T cells are essential and sufficient to establish immediate protection against reinfection with pathogens (20, 41, 42). The remarkable effectiveness of T_RM_ cells to achieve clearance of infection and their potential protective capacities in anti-tumor responses have spurred investigation into the regulatory mechanisms underlying the differentiation, maintenance, and effector functions of these memory CD8 T cells. Transcription factors play important roles in the regulation of memory T cells through their ability to modulate gene expression. Recently, we have identified homolog of Blimp-1 in T cells (Hobit) as a T_RM_-specific transcription factor that together with related Blimp-1 essentially contributes to the differentiation and/or maintenance of T_RM_ cells (43). Besides Hobit and Blimp-1, other factors, including Runx3, Notch, aryl hydrocarbon receptor (Ahr), and NR4A1 are involved in the regulation of T_RM_ cells (Figure 1), suggesting that these cells are under the control of a network of transcription factors (37, 44–46). In this review, we will focus on the role of transcription factors during the different stages of T_RM_ differentiation and during the reactivation of T_RM_ cells upon pathogen re-challenge.

## From Naïve to Memory Cell—Differentiation of T_RM_ Cells

The development of naïve CD8 T cells into effector T cells and subsequently into T_RM_ cells involves priming in the LN, migration from the LN to the peripheral tissues and the acquisition of a T_RM_ phenotype to establish local retention. Here, we will discuss the cell intrinsic signals and tissue-derived cues that instruct the generation and maintenance of T_RM_ cells.

### Heterogeneity in Effector CD8 T Cells—T_RM_ Precursors

The “one cell, multiple fates” hypothesis describes the potential of a single naive CD8 T cell to generate diverse subsets of effector and memory CD8 T cells (47, 48). Studies using genetic barcoding and adoptive transfers of single naïve T cells have demonstrated that T_CM_ and T_EM_ cells can differentiate from the same naïve CD8 T cell. However, it was not addressed whether T_RM_ cells originate from the same naïve T cells as T_CM_ and T_EM_ cells. More recent studies using deep sequencing of the T cell receptor (TCR) β repertoire have revealed substantial overlap in TCR usage between T_CM_ and T_RM_ populations in a skin immunization model (49), suggesting that T_CM_ and T_RM_ cells may develop from a common progenitor. However, given that the naive CD8 T cell population may contain multiple clones bearing identical TCRs, the development of T_CM_ and T_RM_ cells from different precursors cannot be completely excluded.

After recognition of cognate antigen, naïve CD8 T cells first differentiate into effector CD8 T cells. Effector cells diversify into different subsets that include terminal effector cells (TECs) and memory precursor effector cells (MPECs). TECs are characterized by surface expression of killer cell lectin-like receptor G1 (KLRG1) (50). In contrast, memory precursors express very low amounts of KLRG1, but maintain expression of IL-7Rα (CD127) (51). The IL-7Rα^hi^ MPECs differentiate into long-lived memory CD8 T cell populations, whereas the majority of TECs undergoes apoptosis after clearance of the infection. While these studies showed that circulating memory cells develop from MPECs, it was not addressed whether this is the case for T_RM_ cells. Similar to the spleen, peripheral organs such as the skin and small intestine contain KLRG1^+^ and KLRG1^−^ fractions within the virus-specific effector CD8 T cell population after infection (25, 29). The cells that remain within the skin and small intestine at the memory stage lack expression of KLRG1, suggesting that tissue-residing T_RM_ cells develop from MPECs. Indeed, adoptive transfer of the KLRG1^+^ and KLRG1^−^ fractions confirmed that T_RM_ cells preferentially arise from KLRG1^−^ MPECs (25). A regulatory role has been reported for transforming growth factor (TGF) β in controlling TEC cell numbers under acute inflammatory conditions (52). Therefore, local TGF-β signaling may drive the preferential development of MPECs in the small intestine, by selectively inducing apoptosis of the TEC fraction during clonal expansion. Recently, *Klrg1* lineage reporter mice have been developed to track the memory offspring of KLRG1^+^ cells after *Listeria* infection. Fate mapping using the KLRG1 reporter mice showed that approximately half of the T_RM_ cells in the liver and small intestine originate from KLRG1^+^ precursors (53). These findings suggest that the T_RM_ precursor population may contain MPECs that transiently expressed KLRG1 besides MPECs that never expressed KLRG1.

While T_CM_, T_EM_, and T_RM_ cells all appear to develop from MPECs, the timing of branching into the different memory subsets remains unclear. Single cell sequencing data of effector CD8 T cells after the first cell division have revealed only two separate populations that correspond to TECs and MPECs (54), suggesting that at this early stage MPECs form a uniform population. It is conceivable that heterogeneity within MPECs arises at later stages. Adoptive transfer experiments have shown that as early as 7 days after viral infection, effector cells within the spleen have lost the potential to contribute to T_RM_ formation in the intestinal epithelium, while these cells retain the potential to form circulating memory cells (14). These experiments suggest separation between the T_CM_, T_EM_, and T_RM_ lineages at the peak of the effector response. Consistent with this time frame of T_RM_ commitment, kinetic analysis of the upregulation of T_RM_-associated molecules, e.g., CD69 and CD103, during CD8 T cell responses demonstrated that pathogen-specific CD8 T cells within the small intestine and skin acquire a T_RM_ phenotype between 1 and 2 weeks after infection (25, 29, 44, 55). In fact, transcriptional profiling of effector CD8 T cells in the small intestine after lymphocytic choriomeningitis virus (LCMV) infection has shown that the T_RM_-associated program is largely established within 1 week (44).

### Signals Driving T_RM_ Differentiation

Sensing of inflammation and tissue damage during priming of T cells provide important cofactors for the generation of T_RM_ cells. Activated CD8 T cells home to inflamed tissues and can subsequently form T_RM_ cells at these locations, even when antigen is not present locally (41). *In vitro* experiments suggest that inflammatory stimuli may also induce T_RM_ differentiation in the peripheral tissues. Inflammatory cytokines, including type I IFN, IL-33, and tumor necrosis factor-α (TNF-α), downregulate expression of the transcription factor Krüppel-like factor 2 (KLF2) and the tissue exit receptor S1PR1 and upregulate expression of CD69 on CD8 T cells (26, 56). *In vivo* evidence supports such a role for pro-inflammatory cytokines including type I IFN and IL-12 in T_RM_ differentiation (57). Local inflammatory cues might contribute differently to the generation and persistence of mucosal and non-mucosal T_RM_ cells. Inflammatory cytokines such as IFN-β and IL-12 counter-regulate the induction of CD103 by TGF-β during CD8 T cell priming and support the formation and persistence of CD103^−^ CD69^+^ T_RM_ cells in the small intestine (58). Binding of pSTAT4, which can be induced by IL-12 or type I IFN, to the CD103 encoding gene suggests that sensing of inflammation might directly affect CD103 expression (58).

These inflammatory signals might guide T_RM_ generation at different stages of CD8 T cell differentiation, with initial cues for commitment to the T_RM_ lineage already being provided in the lymph node. A specialized population of lymph node residing and crosspresenting CD8α^+^ DCs can provide signals, including IL-12, IL-15, and co-stimulation *via* CD24, which contribute to optimal generation of T_RM_ cells (59). Circulating memory CD8 T cells do not share this requirement for CD8α^+^ DCs in the early stages, suggesting that these DCs specifically drive the formation of T_RM_ cells. Following these early events during priming, effector T cells are recruited to the infected tissue. The inflammatory chemokine receptors CXCR3 and CCR5 have been shown to contribute to the recruitment of T_RM_ precursors. CXCR3 enables T_RM_ precursor cells to respond to the IFN-γ inducible chemokines CXCL9 and CXCL10, which is critical for differentiation of T_RM_ cells in the skin (25). CCR5 ligands provided by pro-inflammatory macrophages are important to instruct recruitment of T_RM_ precursors into the vaginal mucosa (60). These pro-inflammatory signals can be provided by a local network of macrophages (57, 60, 61). Thus, it appears that inflammatory stimuli within the LN and from the local environment contribute to T_RM_ differentiation.

The presence of local antigen is not required to attract activated CD8 T cells into the inflamed tissue (41, 62). In the skin, these activated CD8 T cells can subsequently develop into T_RM_ cells in the absence of local antigen (41). However, T_RM_ cell formation after local skin infection is greatly enhanced in the presence of cognate antigen in the tissue microenvironment (63–65). In other tissues, such as the lung and central nervous system, establishment of T_RM_ cells requires cognate antigen recognition in the tissue (28, 62). In the salivary glands, T_RM_ cell formation depends on antigen in the CD4 T cell compartment, but not in the CD8 T cell compartment (66). The presence of local antigen may, therefore, not impact the size of the effector response in the tissue, but rather promote local retention and the formation of T_RM_ cells. The role of antigen after establishment of T_RM_ cells is less clear, but the long-term maintenance of the T_RM_ cell pool in the lung and small intestine appears to be independent of local antigen (56, 67). Next to antigen, costimulatory signals might contribute to the differentiation of T_RM_ cells. Recent work has demonstrated the requirement of intrinsic signals *via* the tumor necrosis factor (TNF) receptor family member 4-1BB for the generation of influenza-specific CD8 T cells in the lung, in contrast to secondary lymphoid tissues (68).

Next to inflammation and local antigen, the accompanying tissue damage might also contribute to T_RM_ generation. Immunization *via* skin scarification generates highly protective T_RM_ cells, compared to subcutaneous or intradermal injection (69) and lung-resident T cells localize at spots that show signs of recovery from previous tissue damage (70). The factors contributing to these effects are still unknown. Inflammation accompanying tissue damage could be partly responsible for the accumulation of T_RM_ cells at sites of tissue damage. Additionally, competition for survival factors during the reorganization of the tissue after injury might influence T_RM_ persistence (71). Data on the local composition of skin-resident T cells support this view. Pre-existing tissue-resident dendritic epidermal γδ T cells are depleted at sites of infection and are replaced by virus-specific CD8αβ T cells (72). To cope with the inflection-related changes in their microenvironment, T_RM_ cells might have developed tissue-specific adaptations. For example, lung T_RM_ cells constitutively express the interferon-induced transmembrane protein 3 (IFITM3), which facilitates their survival during secondary challenges with influenza (73).

### Maintenance of T_RM_ Cells

Tissue-resident memory CD8 T cells can persist in tissues for long periods of time (13, 20, 57). Their location at distinct sites throughout the body suggests different requirements for their maintenance and specific adaptions to the local environments. The local presence of antigen, cytokines, chemokines, and tissue-specific metabolites are factors that contribute to T_RM_ maintenance.

Similar to recently and chronically activated T cells, T_RM_ cells demonstrate increased expression of activation-associated molecules, such as PD-1 and importantly CD69 (40, 43). However, persistent stimulation by antigen is not required for T_RM_ maintenance. In fact, the development of T_RM_ cells in the intestine is compromised after chronic viral infection compared to acute viral infection (56). In addition, T_RM_ cells can be formed and maintained by recruiting activated T cells into tissues *via* sterile inflammation (41), suggesting that T_RM_ cell persistence does not require local antigen in the peripheral tissues after infection.

Similar to circulating memory cells, T_RM_ cells upregulate receptors for IL-7 and IL-15 (39, 74), suggesting that these homeostatic cytokines contribute to antigen-independent maintenance of T_RM_ cells. Indeed, IL-7 and IL-15 produced within hair follicles maintain T_RM_ cells near these structures within the skin (75). IL-15 already plays a role during lodgment of T_RM_ cells, but the continued presence of IL-15 is essential for long-term T_RM_ maintenance within the skin (74). IL-15 may not be crucial for T_RM_ cells at other sites, as virus-specific T_RM_ cells within the intestines, pancreas, and female reproductive tract (FRT) are maintained independently of IL-15, in contrast to those in the salivary glands and kidneys (76). The involvement of other homeostatic cytokines in the maintenance of these IL-15-independent T_RM_ populations is currently unclear. T_RM_ cells require TGF-β for maintenance in the mucosa (25, 56, 77). TGF-β instructs the upregulation of CD103 that allows retention of T_RM_ cells in the epithelium, potentially through interactions with E-cadherin on epithelial cells (25, 56, 77). TGF-β is produced as part of an inactive complex together with latency associated protein (LAP). Integrins such as α_V_β6 and α_V_β8, which are expressed on keratinocytes, are required to release LAP and activate TGF-β in the epithelium (78). These integrins may restrict the action radius of TGF-β close to the epithelial layer. T_RM_ populations underneath the epithelium such as those within the lamina propria of the intestine are independent of TGF-β and largely do not express CD103 (57). T_RM_ populations within internal organs such as the liver and the kidney also largely lack CD103 expression (17, 43), suggesting TGF-β-independent maintenance. Thus, with notable exceptions, T_RM_ populations are maintained on homeostatic cytokines similar to other memory cells and epithelial T_RM_ cells uniquely require TGF-β.

After development, T_RM_ cells form stable populations in many tissues, including skin, liver, and the small intestine, and provide long-term protection against reinfection (13, 17, 20, 41, 57). Maintenance of T_RM_ cells in these tissues appears to be independent of the recruitment of circulating cells, as adoptive transfer experiments have shown that circulating memory CD8 T cells do not convert into T_RM_ cells under steady state conditions (14). In contrast, influenza-specific T_RM_ cells in the murine lungs fail to survive long-term (67, 79). These T_RM_ cells appear to be continuously replenished *via* recruitment from the circulating memory CD8 T cell pool (67).

Tissue-resident memory CD8 T cells are present throughout the body at distinct sites in highly diverse environments that differ in oxygen and nutrient levels, exposure to microbiota, and the regenerative ability of the tissue. Given that T_RM_ cells are permanently residing within the peripheral tissues, they are strictly dependent on the resources within the local environment in contrast to circulating memory cells. Therefore, T_RM_ cells may require tissue-specific adaptations to cope with different conditions posed by the local microenvironment. Transcriptional profiling has revealed a T_RM_-specific core signature shared between T_RM_ cells at different locations, including the lungs, liver, intestine, and skin (25, 43). In addition to this core signature, T_RM_ cells at different sites are characterized by tissue-specific gene expression profiles (25, 43). The distinct gene programs of T_RM_ cells include chemokine receptors and adhesion molecules that are required to address T_RM_ cells to different tissues. The chemokine receptors CCR8 and CCR10 and the adhesion molecule cutaneous lymphocyte antigen (CLA) are specifically upregulated on skin T_RM_. CCR10 and CLA have also been functionally implicated in the localization of T_RM_ in the skin (25, 80). In contrast, CCR9 is specifically expressed on intestine-derived T_RM_ cells and may, together with the α4β7 integrin, drive localization of T_RM_ cells in the small intestine (14). Skin-resident T_RM_ cells have been described to rely on the uptake of exogenous fatty acids *via* the fatty acid binding protein (FABP) 4 and FABP5 in contrast to circulating memory CD8 T cells (81). The metabolic requirements of T_RM_ cells at other locations are not yet clear. Members of the FABP family are expressed in a tissue-specific manner (82), suggesting that populations within brain, liver, and intestine may take advantage of local opportunities to meet metabolic demands. Thus, the heterogeneity within T_RM_ populations at different locations may reflect strategies to optimally adapt to the local circumstances.

## Effector Responses of T_RM_ Cells upon Reactivation

Numerous studies have highlighted the essential role of T_RM_ cells in providing efficient protection against local reinfections at barrier sites (20, 41, 42). Being situated at the front lines of the immune defense, T_RM_ cells are poised for early detection of recurring pathogens. Here, we will discuss the mechanisms by which T_RM_ cells protect against local infections and the fate of T_RM_ cells after antigen re-encounter.

### Border Patrol

**Figure 2:**
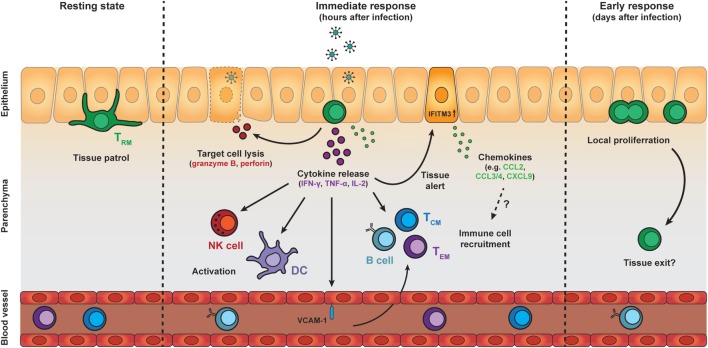
Protective effector responses of epithelial T_RM_ cells upon secondary infection. T_RM_ cells in the epithelia continually patrol their local environment, projecting dendritic extensions in multiple directions. Upon pathogen challenge and antigen re-encounter, T_RM_ cells rapidly release pro-inflammatory cytokines, including IFN-γ, TNF-α, and IL-2, which induce several immune cell- and tissue-specific effects. Local cytokine release by T_RM_ cells results in recruitment and activation of natural killer (NK) cells and dendritic cells (DCs), as well as upregulation of VCAM-1 on endothelial cells in local blood vessels, which may enhance the recruitment of T_CM_, T_EM_, and B cells from the circulation. T_RM_ cell reactivation and cytokine release also induces a tissue-wide state of alert, resulting in upregulation of many innate immune response genes, including interferon-induced transmembrane protein 3 (IFITM3), and the increased local expression of inflammatory chemokines. The protective capacity of T_RM_ cells may also rely on perforin-mediated killing of target cells. One to two days after antigen re-encounter, T_RM_ cells undergo local proliferation. Further investigation is required to determine whether T_RM_ cells exit their local environment after reactivation. Abbreviations: IFN-γ, interferon γ; TNF-α, tumor necrosis factor α; IL-2, interleukin 2; ICAM-1, vascular cell adhesion molecule 1; T_CM_ cell, central memory T cell; T_EM_ cell, effector memory T cell; CCL, C-C motif chemokine; CXCL9, C-X-C motif chemokine 9.

Despite their inability to recirculate throughout the body, T_RM_ cells retain the ability to migrate within their local environment. This has been most extensively studied for T_RM_ cells in the skin. These T_RM_ cells localize to the basal layer of the epidermis, where they migrate in the two-dimensional plane of the tissue. Skin T_RM_ cells display a dynamic morphology and continually project dendritic extensions in multiple directions (72, 83, 84) (Figure 2). In contrast, T cells in the underlying dermis exhibit an amoeboid shape, which resembles that of migrating lymphocytes in the secondary lymphoid organs. The migration of T_RM_ cells within the epidermis appears to be constrained by the local environment upon resolution of inflammation (72). These constraints only permit relatively slow migration of skin T_RM_ cells, thus promoting their long-term persistence at sites of prior infection (72), and enhancing their ability to scan the local environment for recurring pathogens. This local border patrol requires a density of T_RM_ cells of approximately 100 or more cells per mm^2^ for complete coverage of the local area and to ensure early detection of cognate antigens (84).

Patrol of the local tissue environment by T_RM_ cells has also been demonstrated in other organs, e.g., in the FRT and in the liver (17, 18, 85). T_RM_ cells in these tissues show a higher motility compared to the epidermis, which may be related to the more relaxed constraints posed by the tissue architecture. In fact, the speed of T_RM_ cell migration in the FRT is dependent on the local collagen density (85). Local encounter of T_RM_ cells with their cognate antigen in the skin and FRT results in motility arrest and loss of their dendritic morphology (85, 86) (Figure 2). The immobilization is transient and T_RM_ cells resume their migratory behavior within 48 h after antigen re-encounter. Motility arrest upon antigen encounter is important for T cell activation. The transient stop allows for the formation of an immunological synapse between T cells and antigen-presenting cells, and enables T cells to acquire of signals for activation (87). Given that most non-lymphoid tissues are primarily surveyed by T_RM_ cells (21), border patrol by these memory cells likely plays an essential role for the local protection throughout the body. This property as motile sentinels places T_RM_ cells in the front lines of defense, enabling rapid responses to reinfection.

### Early Effector Response of T_RM_ Cells Upon Reactivation

Tissue-resident memory CD8 T cells are among the first immune cells to act in response to pathogens that have been previously encountered in an antigen-specific manner. Upon activation, T_RM_ cells rapidly respond by the production of pro-inflammatory cytokines, including IFN-γ (Figure 2). In both mice and men, T_RM_ cells across different tissues express high transcript levels of these pro-inflammatory cytokines compared to their circulating counterparts (37, 40, 43, 88). These elevated transcript levels may endow T_RM_ cells with the potential to rapidly produce cytokines upon activation. In addition, posttranscriptional mechanisms have been shown to control cytokine production in CD8 T cells (89, 90), and may contribute to the fast responsiveness of T_RM_ cells. IFN-γ has direct antiviral properties, but is also important for the recruitment and activation of immune cells. The early release of IFN-γ by T_RM_ cells has been demonstrated to stimulate immune cells including DCs and NK cells (91). T_RM_-derived IFN-γ also elevates expression of the homing molecule vascular cell adhesion molecule 1 on endothelial cells, and enhances the recruitment of circulating B cells and memory T cells from the bloodstream (60, 91, 92) (Figure 2). Furthermore, antigen recognition by T_RM_ cells potentiates the local expression of inflammatory chemokines in the tissue, including CCL2, CCL3, CCL4, CCL5, CXCL9, and CXCL10 (60, 91). High transcript levels of CCL3, CCL4, and XCL1 in quiescent T_RM_ cells suggest that T_RM_ cells participate themselves in the production of these chemokines (43, 88). T_RM_-derived IFN-γ may also contribute to the release of IFN-γ-dependent chemokines, such as CXCL9 and CXCL10, from the surrounding tissue. These chemokines may trigger the attraction of innate myeloid cells, e.g., neutrophils and monocytes, to the site of infection, thereby further enhancing the immune response (93, 94). In addition, IFN-γ release by reactivated T_RM_ cells has been shown to induce a tissue-wide state of alert in the skin, resulting in elevated expression of many innate immune response genes, including IFITM3, in the tissue (95) (Figure 2). Under certain conditions, T_RM_ cells may even induce a body-wide state of alert to prevent viral spread (96). Interestingly, while the local activation of T_RM_ cells is pathogen-specific, the triggering of downstream immune responses can ultimately lead to near-sterile protection of the tissue against antigenically unrelated pathogens (92, 95). The importance of cytokine production by T_RM_ cells for tissue protection has also been demonstrated in the lung, where airway T_RM_ cells protect against respiratory influenza virus through production of IFN-γ (42). Similarly, IFN-γ production by brain T_RM_ cells is crucial for protection against intracerebral infections (97). Tissue-specific adaptations may exist in the secreted factors of T_RM_ cells at different locations (96). For example, lung-resident T_RM_ cells release IL-22 next to IFN-γ, while T_RM_ cells in the liver co-produce granulocyte-macrophage colony-stimulating factor and IFN-γ (96). These differences in local cytokine repertoires may allow T_RM_ cells to tailor responses to their local microenvironment.

Protection against intracellular pathogens by effector CD8 T cells is partly mediated by the removal of infected cells through the targeted release of cytotoxic molecules, including perforin and granzyme B. After clearance of infection, the expression of cytotoxic molecules is strongly downregulated in circulating memory CD8 T cells. In contrast, T_RM_ cells in several tissues maintain high levels of granzyme B in the memory phase (17, 56, 97) (Figure 1). The constitutive expression of granzyme B suggests that T_RM_ cells can rapidly employ cytotoxic mechanisms to eliminate infected cells early after pathogen re-encounter. Indeed, T_RM_ cells in the brain can kill target cells and their protective capacity is dependent on perforin (28, 97). Granzyme B has furthermore been implicated in the remodeling of extracellular matrices (98, 99), suggesting that the serine protease may also contribute to the local migration of T_RM_ cells within tissues. Granzyme B-driven cytotoxicity may not be essential for T_RM_-mediated protection at other sites, given that, for example, airway T_RM_ cells do not maintain expression of granzyme B and other cytotoxic mediators (42). The selective killing of infected cells by T_RM_ cells minimizes off-target immunopathology, but this protective mechanism may be overwhelmed by rapidly replicating pathogens. Under these conditions, the potential of T_RM_ cells to amplify immune responses through the release of pro-inflammatory cytokines and chemokines may be essential and offset the increased risk for collateral damage.

### Proliferation and Maintenance of the Local T_RM_ Repertoire

The protective capacity of memory CD8 T cells depends on their robust proliferation upon recall to establish an army of secondary effector cells. The large number of effector cells can be crucial to counter rapidly replicating and spreading pathogens. In particular, T_CM_ cells have a robust proliferative capacity (100–102). These memory cells patrol secondary lymphoid organs and are, therefore, ideally positioned at these distal sites to the infection to mount secondary responses. T_EM_ cells, which survey peripheral tissues and have limited access to the LN, undergo less pronounced proliferation upon re-challenge (100–102). Using intravital imaging, it has been demonstrated that T_RM_ cells in the skin and FRT undergo local proliferation *in situ* within the first days after antigen re-encounter (85, 86). Potential changes in phenotypic markers on reactivated T_RM_ cells and timespan limitations for intravital imaging pose challenges for long-term follow-up of secondary T_RM_ responses. Despite these technical difficulties, it appears that pre-existing T_RM_ cells within peripheral tissues are the main origin of local proliferative recall responses (Figure 2). In line with this, the secondary T_RM_ population arising after pathogen clearance primarily develops from pre-existing T_RM_ cells (85, 86). Recruited circulating memory CD8 T cells also contribute to secondary effector responses (68) and the formation of secondary T_RM_ cells, albeit to a lesser extent (85, 86). However, these memory cells appear to have a limited potential to form T_RM_ cells, at least compared to naïve CD8 T cells (103). The importance of the recruitment of circulating memory cells into the secondary T_RM_ pool may reside in the introduction of new specificities to the local repertoire. Despite local proliferation, reinfection does not numerically increase the pool of local T_RM_ cells (86), suggesting that limits exist in the number of T_RM_ cells that can populate the peripheral tissues. If that is indeed the case, then secondary T_RM_ cells may compete for available niches, which may re-shape the local repertoire after reinfection (71). Previously, it has been demonstrated that circulating memory T cells undergo qualitative changes after successive infections (104, 105). In this context, it will be interesting to investigate the quality, function, and longevity of these secondary T_RM_ cells compared to primary T_RM_ cells.

### Tissue Exit and Contribution to Systemic Responses

While local reinfection results in the recruitment of circulating memory T cells to the tissue, locally proliferating T_RM_ cells may in turn downregulate their tissue residency program and egress from the peripheral tissues. Secondary lymphoid organs, including lymph nodes (LN) that drain tissues, are mainly populated by circulating naïve and memory T cells, but also harbor T_RM_ cells (34). Recent work has shown that the T_RM_ cell population in the draining LN increases after a secondary challenge in the skin or the FRT and that these secondary T_RM_ cells are derived from reactivated T_RM_ cells in the non-lymphoid tissue (22). This demonstrates that, upon antigen exposure, T_RM_ cells possess the ability to leave their local environment and enter other tissues, where they can form secondary T_RM_ cells. It remains to be determined whether T_RM_ cells can also disseminate beyond the local draining LN and form secondary memory cells in anatomically distinct tissues (Figure 2). Consistent with a contribution of T_RM_ cells to systemic secondary responses, adoptively transferred intestinal T_RM_ cells can acquire properties of circulating memory CD8 T cells upon re-stimulation (55). Further work is required to address whether *in situ* reactivated T_RM_ cells also differentiate into circulating effector and memory cells during secondary responses. After tissue exit, reactivated T_RM_ cells may return to their tissue of origin. Previous work has demonstrated that re-stimulated memory CD8 T cells have a homing bias to their tissue of origin (27, 106), suggesting that reactivated T_RM_ cells may retain an imprint that permits re-entry into their former tissue of residence.

## Transcriptional Control of T_RM_ Differentiation and Function

The transition of naïve CD8 T cells into effector and memory cells is a tightly coordinated differentiation process under the control of transcription factors. Upon activation, naïve CD8 T cells upregulate a transcriptional program that drives their differentiation into effector CD8 T cells, thus enabling the establishment of immune responses against pathogens. After clearance of infection, T_CM_ and T_EM_ cells downregulate the effector program and partially re-acquire transcriptional regulators of naïve CD8 T cells to assist in the long-term maintenance of these memory CD8 T cells. In contrast to circulating memory T cells, T_RM_ cells retain immediate potential to exert effector functions and do not re-establish body-wide immune-surveillance. Therefore, growing evidence suggests that T_RM_ cells require a specific program of transcriptional regulation. Here, we summarize data on the role of T_RM_ cell-specific transcription factors as well as on how transcription factors with a crucial role for effector CD8 T cell differentiation regulate T_RM_ cell generation and maintenance. Finally, we will discuss the transcriptional regulation of T_RM_ effector function and T_RM_ differentiation upon activation in secondary responses.

### Transcription Factors Regulating Tissue Residency

**Figure 3:**
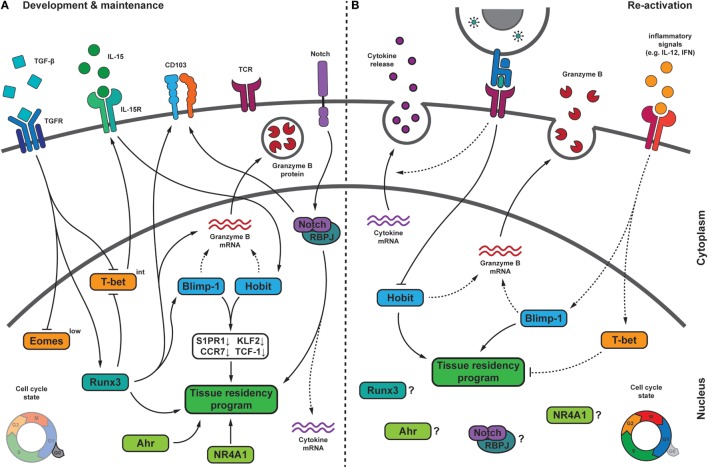
Transcriptional regulation of T_RM_ cells during development, maintenance, and upon pathogen re-challenge. **(A)** During their formation, T_RM_ cells receive multiple signals from the tissue microenvironment that integrate into a transcriptional program, which drives T_RM_ differentiation and maintenance. In several tissues, transforming growth factor β (TGF-β) signaling drives the downregulation of the T-box transcription factors Eomesodermin (Eomes) and T-bet. Residual T-bet expression is required for IL-15Rβ expression. The transcription factor homolog of Blimp-1 in T cells (Hobit) can be induced in an IL-15-dependent manner and, together with its homolog Blimp-1, represses the expression of S1PR1, CCR7, Krϋpple-like factor 2 (KLF2), and TCF-1, which is crucial for tissue residency. Blimp-1 and Hobit may also contribute to granzyme B maintenance in T_RM_ cells. The transcription factor Runx3, which can be induced by TGF-β signaling, is crucial for the establishment and maintenance of many aspects of T_RM_ cells, including granzyme B and CD103 expression. Runx3 has been shown to induce Blimp-1. Notch may regulate expression of the adhesion molecule CD103, is essential for maintenance of T_RM_ cells and might contribute to the elevated transcript levels encoding for pro-inflammatory cytokines in T_RM_ cells. Other factors regulating T_RM_ cells include the nuclear receptor NR4A1, and the aryl hydrocarbon receptor (AhR). During quiescence, T_RM_ cells show low proliferative activity. **(B)** Following pathogen re-encounter, T_RM_ cells are exposed to antigen-dependent T cell receptor (TCR) triggering and a variety of inflammatory signals. TCR triggering in T_RM_ cells may result in downregulation of Hobit, thereby weakening its contribution to maintenance of tissue residency. Inflammatory signals, such as IL-12 and type I interferons (IFN), can induce expression of Blimp-1 and T-bet. While increased Blimp-1 expression might fortify T_RM_ features, elevated levels of T-bet could interfere with tissue residency. T_RM_ cells rapidly release pro-inflammatory cytokines upon reactivation in an antigen-dependent manner. Upon re-infection, T_RM_ cells undergo local proliferation. Dashed lines indicate relations that require further investigation. Abbreviations: Blimp-1, homolog of B lymphocyte-induced maturation protein 1; IL, interleukin; S1PR1, sphingosine-1-phosphate receptor 1; CCR7, C-C chemokine receptor type 7; TCF-1, T cell factor 1; Runx3, Runt-related transcription factor 3; RBPJ, recombining binding protein suppressor of hairless; NR4A1, nuclear receptor subfamily 4 group A 1.

Gene expression analysis of circulating memory CD8 T cells and T_RM_ cells has revealed transcription factors with T_RM_-restricted expression profiles (Figure 3). One of these T_RM_ -specific transcription factors is Hobit. Hobit is upregulated in murine T_RM_ cells within skin, lungs, liver, kidney, small intestine, and brain, suggesting that Hobit is widely expressed throughout T_RM_ populations (25, 43, 88). These Hobit^+^ T_RM_ populations include CD103^+^ T_RM_ cells within epithelial tissues and CD103^−^ T_RM_ cells within internal organs, underlining that the transcription factor is ubiquitously expressed in murine T_RM_ subsets. In addition, other tissue-resident lymphocytes such as natural killer T (NKT) cells and innate lymphoid cells 1 express Hobit, suggesting that Hobit is a central regulator of the tissue-residency program of lymphocytes (43). Due to limitations in access to peripheral tissues, analyses of Hobit expression in human T_RM_ cells have not been as extensive as in mice. In line with findings in mice, a substantial proportion of CD69^+^ CD8 T cells within the human liver expresses Hobit at the protein level (38, 107). Transcriptional profiling also revealed that CD69^+^ CD8 T cells in human lungs express Hobit in contrast to their CD69^−^ counterparts, although expression levels are low compared to murine T_RM_ cells (40). We have previously described that CD45RA^+^ CD27^−^ effector and CD45RA^−^ CD27^−^ effector memory CD8 T cells in human peripheral blood also express Hobit (108). Therefore, despite the presence of Hobit in subpopulations of human T_RM_ cells, no strict association of Hobit with tissue residency exists in human CD8 T cells.

In mice, Hobit specifically instructs the differentiation and/or maintenance of T_RM_ cells, but the transcription factor does not operate alone. Hobit is highly homologous to Blimp-1 and both factors co-operate in the transcriptional regulation of T_RM_ cells. Hobit and Blimp-1 both recognize a “GAAAG” containing binding motif and share the majority of their DNA-binding sites, suggesting that the related factors collaborate through competitive regulation at overlapping target genes. Hobit and Blimp-1 lock T_RM_ cells into the tissues, as these transcription factors instruct shutdown of exit pathways through CCR7 and S1PR1, thus preventing T_RM_ cells from re-entering the circulation (43). In circulating memory cells, the transcription factor KLF2 drives the expression of S1PR1 to provide access to the blood or lymph (109–111). Downregulation of S1PR1 and KLF2 is essential for T_RM_ differentiation, as evidenced by forced expression of S1PR1 that completely prevents the generation of T_RM_ cells (26). The Wnt signaling associated transcription factor TCF1 is involved in maintenance of the distinct phenotype of T_CM_ cells, including upregulation of CD62L and CCR7 (112). Hobit and Blimp-1 directly bind within the *Klf2* and the TCF1 encoding *Tcf7* locus and within the loci of the downstream targets S1PR1 and CCR7, suggesting that these transcription factors efficiently downregulate tissue exit pathways at multiple levels (26, 43) (Figure 3). The expression of Hobit in circulating human effector-type and effector memory-type CD8 T cells is enigmatic, given that Hobit in mice directly suppresses expression of tissue exit receptors. Although S1PR1 and CCR7 are nearly absent in quiescent human effector CD8 T cells (113), the putative repressive actions of Hobit on these pathways in long-lived human effector CD8 T cells appear insufficient to retain these cells within the peripheral tissues.

Other T_RM_-specific transcription factors contribute to the regulation of T_RM_ cells. Expression of the Ahr has been identified in T_RM_ populations of the lungs, skin, and small intestine, but not in circulating memory CD8 T cells (25). In line with its expression pattern, Ahr specifically regulates the persistence of T_RM_ in the skin after HSV infection (72). Ahr is a ligand-operated transcription factor that responds to the presence of dietary components (45), but its ligands in virus-specific T_RM_ cells within the skin are unknown. The transcription factor NR4A1 is also expressed in T_RM_ cells in contrast to circulating memory CD8 T cells (46). NR4A1 is specifically involved in the development and/or maintenance of T_RM_ populations, in particular those in the epithelium and lamina propria of the small intestine (46). The downstream targets of Ahr and NR4A1 in T_RM_ cells have not been identified. Therefore, it remains unclear which aspects of T_RM_ differentiation are regulated by these transcription factors.

### Regulation of T_RM_ Cells by Transcription Factors of Effector CD8 T Cells

Runx3, T-bet, Blimp-1, and Notch are each individually important in driving terminal differentiation of effector CD8 T cells and in the acquisition of important effector functions including the production of IFN-γ and/or cytotoxicity (50, 114–117). T_RM_ cells maintain direct effector function into the memory phase, suggesting a requirement for the persistent activity of these transcription factors. Indeed, Runx3, T-bet, Blimp-1, and Notch have also been implicated in the development and/or in the maintenance of T_RM_ cells (37, 43, 44, 74) (Figure 3).

Runx3 drives the generation of the CD8 T cell lineage in the thymus and is broadly expressed in peripheral naïve, effector, and memory CD8 T cells (118, 119). Runx3 pairs with the obligatory factor core binding factor of the Runx family that stabilizes binding of Runx proteins, including Runx3, to DNA (120). Functional profiling of CD8 T cell responses demonstrated that Runx3 expression is more relevant in T_RM_ cells than in circulating memory CD8 T cells (44). The transcriptional activity of Runx3 is already apparent at the effector stage in putative T_RM_ precursors, suggesting that Runx3 drives the formation of T_RM_ cells. Runx3 remains essential during the memory phase, implicating a continued role for Runx3 in the maintenance of T_RM_ cells (44). Virus-specific and tumor-specific T_RM_ cells in different tissues and settings require Runx3 for development, exemplifying Runx3 as an important transcriptional regulator of T_RM_ cells. Overexpression of Runx3 is sufficient to repress the expression of signature genes of circulating memory CD8 T cells and to promote the expression of residency signature genes including that of CD103 (44, 121). Collectively, these observations suggest that Runx3 has a primary role in the transcriptional regulation of T_RM_ differentiation. Runx3 may act upstream of Hobit and Blimp-1 in T_RM_ cells, given that the transcription factor induces expression of Blimp-1 and enhances accessibility to motifs shared by Hobit and Blimp-1 (122).

Notch is a surface receptor that interacts with the membrane-bound ligands Jagged and Delta-like on antigen-presenting cells (123). After ligand-induced activation, Notch is cleaved by γ-secretase, which enables its intracellular domain to translocate to the nucleus. Following translocation, Notch associates with the DNA-binding factor recombining binding protein suppressor of hairless (RBPJ) to form a transcriptional activator (124). Notch signaling orchestrates the maintenance of CD103^+^ T_RM_ cells in the lungs after influenza infection (37). TGF-β-driven upregulation of Notch ligands within the epithelium may provide a mechanism to activate Notch specifically at these sites (125, 126). Notch appears to directly regulate expression of CD103 (37), thus facilitating binding of T_RM_ cells within the epithelium. In addition, downstream targets of Notch include the glycerol transporter aquaporin-3, solute carriers for amino acids and other nutrients, suggesting that Notch contributes to the maintenance of T_RM_ cells through regulation of their metabolism (37).

T-bet is a T-box factor family member, which drives expression of the IL-15 receptor in circulating memory CD8 T cells (127) and appears to have a comparable role in T_RM_ cells (74). Similar to circulating memory CD8 T cells, T_RM_ populations in several, but not all tissues, require the homeostatic cytokine IL-15 for long-term maintenance (74, 76). Underlining its subordinate role in T_RM_ cells, it has been reported that T-bet acts at a lower level of expression in T_RM_ cells than in effector or circulating memory CD8 T cells. T_RM_ cells also completely lack the T-bet-related T-box factor Eomesodermin (Eomes) that, similarly to T-bet, can support IL15 receptor expression in circulating memory CD8 T cells (74). Overexpression of T-bet or Eomes abrogates differentiation of T_RM_ cells in skin and lungs, suggesting that high-level expression of these transcription factors is incompatible with long-term survival of T_RM_ cells (74, 128). The expression of T-bet is suppressed in T_RM_ cells in a TGF-β- and Runx3-dependent manner (44, 74). Downregulation of T-bet may dampen its suppressive impact on the CD103 encoding *Itgae* locus, where T-bet is able to bind at sites that overlap with the TGF-β-driven Smad proteins (128). Therefore, reduction of T-bet expression may limit interference with TGF-β-driven induction of CD103 expression in T_RM_ cells, while the residual T-bet expression may be sufficient to upregulate IL-15 receptor in T_RM_ cells and to receive IL-15-dependent survival signals for homeostasis (128).

Taken together, transcriptional regulation of T_RM_ cells includes the up-regulation of T_RM_-specific transcription factors, suppression of transcription factors important for circulating memory T cells, and the maintenance of transcription factors involved in effector differentiation.

### Transcriptional Regulation of Direct Effector Functions of T_RM_ Cells

Tissue-resident memory CD8 T cells rapidly exert effector functions upon activation, suggesting that transcription factors that regulate the expression of cytotoxic and pro-inflammatory molecules may also be active in T_RM_ cells. Interestingly, transcription factors that are important for T_RM_ development also play crucial roles in the regulation of effector functions.

In contrast to circulating memory T cells, T_RM_ cells maintain expression of the cytotoxic mediator granzyme B at the protein level, which provides them with the potential to contain infection at early stages through the elimination of infected cells. Runx3 has been shown to induce expression of granzyme B in T_RM_ cells, directly implicating the transcription factor in the regulation of cytotoxicity in these memory T cells (44). A role for Runx3 in the instruction of lytic activity through the upregulation of granzyme B and perforin expression has been previously established in effector CD8 T cells (114, 129). Runx3 directly binds at the granzyme and perforin loci, but also recruits Eomes for synergistic activity at the perforin locus in effector CD8 T cells (114). Mucosal T_RM_ cells do not express Eomes (74), suggesting that in these cells the activity of Runx3 is Eomes-independent. The Runx3-driven program of cytotoxicity in effector CD8 T cells may also involve the upregulation of Blimp-1 expression (129). Blimp-1 and its homolog Hobit have been directly implicated in the regulation of cytotoxicity in effector CD8 T cells (115, 116) and in NKT cells (130), respectively. Blimp-1 drives the acquisition of granzyme B in effector CD8 T cells after acute infection with LCMV and influenza (115, 116). Hobit is required for NKT cells to upregulate granzyme B after stimulation with pro-inflammatory cytokines such as type I IFN and after infection with mCMV (130). The role of Hobit and Blimp-1 in the regulation of cytotoxicity in T_RM_ cells remains to be investigated. The transcriptional regulation of cytotoxicity in T_RM_ cells involves the long-term maintenance of cytotoxic molecules during steady state. Currently, it is not clear how the transcriptional network of T_RM_ cells achieves the retention of cytotoxic molecules into the memory phase. Constitutive expression of Runx3, Blimp-1, and Hobit in T_RM_ cells may be required for persistent expression of granzyme B and other cytotoxic molecules in these memory T cells (Figure 3).

Tissue-resident memory CD8 T cells are able to mount rapid cytokine responses upon reactivation, which at least in part resides in their superior capacity to retain mRNA molecules encoding pro-inflammatory cytokines, including IFN-γ (43, 88). The transcriptional network underlying the persistence of mRNA of pro-inflammatory cytokines has not yet been established. Important transcriptional regulators of IFN-γ include T-bet and Eomes (127, 131), but these T-box transcription factors are downregulated in T_RM_ cells in mice and humans (37, 74, 128), suggesting that they do not play a dominant role in T_RM_ cells. Runx3 has been described to regulate IFN-γ, TNF-α, and IL-2 in effector CD8 T cells (114), but is not essential for the regulation of cytokine production by T_RM_ cells (44). Although Notch ligands induce IFN-γ expression in human T_RM_ cells, Notch deficiency only marginally reduces the expression of IFN-γ in murine T_RM_ cells (37). It is possible that the absence of an essential role in the regulation of IFN-γ production for any of these transcription factors relates to redundancy between the IFN-γ-driving molecules.

Taken together, the overlap in the transcriptional programs of effector CD8 T cells and T_RM_ cells suggest a high degree of conservation in the regulation of their effector capacities. Understanding the interplay between the different transcriptional programs in the maintenance of the poised effector state of T_RM_ cells is crucial to further unravel the underlying transcriptional network.

### Transcriptional Regulation of T_RM_ Cells Upon Re-Stimulation

While the transcriptional program of T_RM_ generation and maintenance is starting to become clear, it is currently not known how transcription factors regulate T_RM_ functions after reactivation during reinfection. Based on the available information in circulating CD8 T cells, we can speculate on how the signals received by T_RM_ cells during infection may influence their transcriptional program (Figure 3).

The transcription factor Hobit is specifically expressed by T_RM_ cells and other tissue-resident lymphocytes including NKT cells during quiescence. Antigen recognition by NKT cells leads to immediate downregulation of Hobit (130). Hobit expression might be similarly regulated in T_RM_ cells. Downregulation of Hobit after TCR activation might allow T_RM_ cells to release effector molecules and undergo proliferation. Additionally, the loss of the tissue-residence transcription factor Hobit might enable T_RM_ cells to leave the tissue, enter the circulation, and migrate to secondary lymphoid organs. In memory CD8 T cells, the sensing of inflammation alone without cognate antigen recognition is sufficient to induce upregulation of effector molecules such as granzyme B (132). IFN-α receptor 1 and signal transducer and activator of transcription 1 are critical in this bystander cytotoxicity of circulating memory CD8 T cells. In NKT cells, Hobit is crucial for the ability to respond to inflammatory cytokines and type I interferon-driven granzyme B upregulation (130). Similarly, Hobit expression may also drive the innate functions of T_RM_ cells after recognition of inflammation.

As pointed out above, many of the transcription factors, which are induced during priming of naïve CD8 T cells and upregulated in effector cells, are also critical for T_RM_ formation and maintenance. Blimp-1 and T-bet are highly expressed in effector T cells and maintained at a lower level in memory CD8 T cells (50, 115, 116). Upon reinfection, reactivated memory cells form secondary effector cells that phenotypically and transcriptionally resemble primary effector cells, e.g., high expression of T-bet. Recognition of IL-12 by memory CD8 T cells during recall responses is one of the main drivers of T-bet upregulation (133). Blimp-1 expression may be similarly regulated, as Blimp-1 is induced by pro-inflammatory cytokines including IL-12 *in vitro* (134). The data suggest that Blimp-1 and T-bet are upregulated in T_RM_ cells in response to inflammation and/or TCR triggering. Given its crucial role in T_RM_ differentiation, increased expression of Blimp-1 may manifest tissue-resident features upon reinfection. At the same time, concurrent inflammation-induced upregulation of T-bet may interfere with maintenance of tissue residency, as elevated levels of T-bet are incompatible with T_RM_ formation (74). The role of the transcription factors Ahr and NR4A1 during activation of memory CD8 T cells is less clear. The expression of Ahr is increased upon activation of memory T cells (135). Also NR4A1 expression is upregulated after TCR triggering (136), but appears to exert a regulatory role after activation, as the transcription factor can maintain T cells in a quiescent state *via* the suppression of IRF4 (137). These data suggest that changes in the transcriptional programming of T_RM_ cells likely occur upon reactivation. Further research is required to determine how the transcriptional network of T_RM_ cells controls their function and differentiation upon re-challenge with antigen and/or inflammation during infection.

## Concluding Remarks

The unique properties of T_RM_ cells compared to circulating memory CD8 T cells have sparked interest in the development of therapeutic approaches that induce T_RM_ formation, especially in the context of future vaccination strategies (138, 139). Given their superior protective capacity at barrier sites, local establishment of T_RM_ cells constitutes an attractive approach to confer long-lasting tissue immunity. Recent work has demonstrated the potency of vaccine-induced T_RM_ cells in providing protection against heterotypic viral challenges (140) and local tumor development (141, 142). In line with this, the improved survival rates of patients with tumors containing large quantities of T_RM_-type cells highlights T_RM_ cells as a potential target in the treatment of cancer (143–145). A better understanding of the transcriptional network underlying the differentiation and function of T_RM_ cells may assist in unlocking these potent memory cells for therapeutic purposes.

## Author Contributions

FB, AC, RS and KG drafted and edited the manuscript. FB drafted and edited the figures and figure legends. All authors approved the work for publication.

## Conflict of Interest Statement

The authors declare that the research was conducted in the absence of any commercial or financial relationships that could be construed as a potential conflict of interest.

## References

[1] Blattman JN Antia R Sourdive DJD Wang X Kaech SM Murali-Krishna K Estimating the precursor frequency of naive antigen-specific CD8 T cells. J Exp Med (2002) 195(5):657–64. 10.1084/jem.20001021 11877489 PMC2193761

[2] Bousso P Casrouge A Altman JD Haury M Kanellopoulos J Abastado J-P Individual variations in the murine T cell response to a specific peptide reflect variability in naive repertoires. Immunity (1998) 9(2):169–78. 10.1016/S1074-7613(00)80599-3 9729037

[3] Casrouge A Beaudoing E Dalle S Pannetier C Kanellopoulos J Kourilsky P Size estimate of the αβ TCR repertoire of naive mouse splenocytes. J Immunol (2000) 164(11):5782–7. 10.4049/jimmunol.164.11.5782 10820256

[4] van Heijst JWJ Gerlach C Swart E Sie D Nunes-Alves C Kerkhoven RM Recruitment of antigen-specific CD8+ T cells in response to infection is markedly efficient. Science (2009) 325(5945):1265–9. 10.1126/science.1175455 19729659

[5] Butz EA Bevan MJ. Massive expansion of antigen-specific CD8+ T cells during an acute virus infection. Immunity (1998) 8(2):167–75. 10.1016/S1074-7613(00)80469-0 9491998 PMC2776648

[6] Williams MA Bevan MJ. Effector and memory CTL differentiation. Annu Rev Immunol (2007) 25(1):171–92. 10.1146/annurev.immunol.25.022106.141548 17129182

[7] Badovinac VP Harty JT CD8+ T-cell homeostasis after infection: setting the ‘curve’. Microbes Infect (2002) 4(4):441–7. 10.1016/S1286-4579(02)01558-7 11932195

[8] Sprent J Tough DF. T cell death and memory. Science (2001) 293(5528):245–8. 10.1126/science.1062416 11452113

[9] Ahmed R Gray D. Immunological memory and protective immunity: understanding their relation. Science (1996) 272(5258):54–60. 10.1126/science.272.5258.54 8600537

[10] Sallusto F Lenig D Förster R Lipp M Lanzavecchia A. Two subsets of memory T lymphocytes with distinct homing potentials and effector functions. Nature (1999) 401(6754):708–12. 10.1038/44385 10537110

[11] Masopust D Vezys V Marzo AL Lefrançois L. Preferential localization of effector memory cells in nonlymphoid tissue. Science (2001) 291(5512):2413–7. 10.1126/science.1058867 11264538

[12] Gerlach C Moseman EA Loughhead SM Alvarez D Zwijnenburg AJ Waanders L The chemokine receptor CX3CR1 defines three antigen-experienced CD8 T cell subsets with distinct roles in immune surveillance and homeostasis. Immunity (2016) 45(6):1270–84. 10.1016/j.immuni.2016.10.018 27939671 PMC5177508

[13] Gebhardt T Wakim LM Eidsmo L Reading PC Heath WR Carbone FR. Memory T cells in nonlymphoid tissue that provide enhanced local immunity during infection with herpes simplex virus. Nat Immunol (2009) 10:524–30. 10.1038/ni.1718 19305395

[14] Masopust D Choo D Vezys V Wherry EJ Duraiswamy J Akondy R Dynamic T cell migration program provides resident memory within intestinal epithelium. J Exp Med (2010) 207(3):553–64. 10.1084/jem.20090858 20156972 PMC2839151

[15] Anderson KG Sung H Skon CN Lefrancois L Deisinger A Vezys V Cutting edge: intravascular staining redefines lung CD8 T cell responses. J Immunol (2012) 189(6):2702–6. 10.4049/jimmunol.1201682 22896631 PMC3436991

[16] Anderson KG Mayer-Barber K Sung H Beura L James BR Taylor JJ Intravascular staining for discrimination of vascular and tissue leukocytes. Nat Protoc (2014) 9(1):209–22. 10.1038/nprot.2014.005 24385150 PMC4428344

[17] Fernandez-Ruiz D Ng WY Holz LE Ma JZ Zaid A Wong YC Liver-resident memory CD8(+) T cells form a front-line defense against malaria liver-stage infection. Immunity (2016) 45(4):889–902. 10.1016/j.immuni.2016.08.011 27692609

[18] McNamara HA Cai Y Wagle MV Sontani Y Roots CM Miosge LA Up-regulation of LFA-1 allows liver-resident memory T cells to patrol and remain in the hepatic sinusoids. Sci Immunol (2017) 2(9):eaaj1996. 10.1126/sciimmunol.aaj1996 28707003 PMC5505664

[19] Klonowski KD Williams KJ Marzo AL Blair DA Lingenheld EG Lefrancois L. Dynamics of blood-borne CD8 memory T cell migration in vivo. Immunity (2004) 20(5):551–62. 10.1016/S1074-7613(04)00103-7 15142524

[20] Jiang X Clark RA Liu L Wagers AJ Fuhlbrigge RC Kupper TS Skin infection generates non-migratory memory CD8+ TRM cells providing global skin immunity. Nature (2012) 483:227–31. 10.1038/nature10851 22388819 PMC3437663

[21] Steinert Elizabeth M Schenkel Jason M Fraser Kathryn A Beura Lalit K Manlove Luke S Igyártó Botond Z Quantifying memory CD8 T cells reveals regionalization of immunosurveillance. Cell (2015) 161(4):737–49. 10.1016/j.cell.2015.03.031 25957682 PMC4426972

[22] Beura LK Wijeyesinghe S Thompson EA Macchietto MG Rosato PC Pierson MJ T cells in nonlymphoid tissues give rise to lymph-node-resident memory T cells. Immunity (2018) 48(2):327–38.e5. 10.1016/j.immuni.2018.01.015 29466758 PMC5828517

[23] Bankovich AJ Shiow LR Cyster JG. CD69 suppresses sphingosine 1-phosophate receptor-1 (S1P1) function through interaction with membrane helix 4. J Biol Chem (2010) 285(29):22328–37. 10.1074/jbc.M110.123299 20463015 PMC2903414

[24] Mackay LK Braun A Macleod BL Collins N Tebartz C Bedoui S Cutting edge: CD69 interference with sphingosine-1-phosphate receptor function regulates peripheral T cell retention. J Immunol (2015) 194(5):2059–63. 10.4049/jimmunol.1402256 25624457

[25] Mackay LK Rahimpour A Ma JZ Collins N Stock AT Hafon ML The developmental pathway for CD103+ CD8+ tissue-resident memory T cells of skin. Nat Immunol (2013) 14(12):1294–301. 10.1038/ni.2744 24162776

[26] Skon CN Lee J-Y Anderson KG Masopust D Hogquist KA Jameson SC. Transcriptional downregulation of S1pr1 is required for the establishment of resident memory CD8+ T cells. Nat Immunol (2013) 14(12):1285–93. 10.1038/ni.2745 24162775 PMC3844557

[27] Hofmann M Pircher H. E-cadherin promotes accumulation of a unique memory CD8 T-cell population in murine salivary glands. Proc Natl Acad Sci U S A (2011) 108(40):16741–6. 10.1073/pnas.1107200108 21930933 PMC3189029

[28] Wakim LM Woodward-Davis A Bevan MJ. Memory T cells persisting within the brain after local infection show functional adaptations to their tissue of residence. Proc Natl Acad Sci U S A (2010) 107(42):17872–9. 10.1073/pnas.1010201107 20923878 PMC2964240

[29] Sheridan Brian S Pham Q-M Lee Y-T Cauley Linda S Puddington L Lefrançois L Oral infection drives a distinct population of intestinal resident memory CD8+ T cells with enhanced protective function. Immunity (2014) 40(5):747–57. 10.1016/j.immuni.2014.03.007 24792910 PMC4045016

[30] Cepek KL Parker CM Madara JL Brenner MB. Integrin alpha E beta 7 mediates adhesion of T lymphocytes to epithelial cells. J Immunol (1993) 150(8):3459–70. 8468482

[31] Cepek KL Shaw SK Parker CM Russell GJ Morrow JS Rimm DL Adhesion between epithelial cells and T lymphocytes mediated by E-cadherin and the αEβ7 integrin. Nature (1994) 372(6502):190–3. 10.1038/372190a0 7969453

[32] Mueller SN Mackay LK. Tissue-resident memory T cells: local specialists in immune defence. Nat Rev Immunol (2016) 16(2):79–89. 10.1038/nri.2015.3 26688350

[33] Sathaliyawala T Kubota M Yudanin N Turner D Camp P Thome JJC Distribution and compartmentalization of human circulating and tissue-resident memory T cell subsets. Immunity (2013) 38(1):187–97. 10.1016/j.immuni.2012.09.020 23260195 PMC3557604

[34] Schenkel JM Fraser KA Masopust D. Cutting edge: resident memory CD8 T cells occupy frontline niches in secondary lymphoid organs. J Immunol (2014) 192(7):2961–4. 10.4049/jimmunol.1400003 24600038 PMC3965619

[35] Ray SJ Franki SN Pierce RH Dimitrova S Koteliansky V Sprague AG The collagen binding α1β1 integrin VLA-1 regulates CD8 T cell-mediated immune protection against heterologous influenza infection. Immunity (2004) 20(2):167–79. 10.1016/S1074-7613(04)00021-4 14975239

[36] Cheuk S Schlums H Gallais Sérézal I Martini E Chiang SC Marquardt N CD49a expression defines tissue-resident CD8+ T cells poised for cytotoxic function in human skin. Immunity (2017) 46(2):287–300. 10.1016/j.immuni.2017.01.009 28214226 PMC5337619

[37] Hombrink P Helbig C Backer RA Piet B Oja AE Stark R Programs for the persistence, vigilance and control of human CD8+ lung-resident memory T cells. Nat Immunol (2016) 17(12):1467–78. 10.1038/ni.3589 27776108

[38] Stelma F de Niet A Sinnige MJ van Dort KA van Gisbergen KPJM Verheij J Human intrahepatic CD69+ CD8+ T cells have a tissue resident memory T cell phenotype with reduced cytolytic capacity. Sci Rep (2017) 7(1):6172 10.1038/s41598-017-06352-3 28733665 PMC5522381

[39] Thome JJ Yudanin N Ohmura Y Kubota M Grinshpun B Sathaliyawala T Spatial map of human T cell compartmentalization and maintenance over decades of life. Cell (2014) 159(4):814–28. 10.1016/j.cell.2014.10.026 25417158 PMC4243051

[40] Kumar BV Ma W Miron M Granot T Guyer RS Carpenter DJ Human tissue-resident memory T cells are defined by core transcriptional and functional signatures in lymphoid and mucosal sites. Cell Rep (2017) 20(12):2921–34. 10.1016/j.celrep.2017.08.078 28930685 PMC5646692

[41] Mackay LK Stock AT Ma JZ Jones CM Kent SJ Mueller SN Long-lived epithelial immunity by tissue-resident memory T (TRM) cells in the absence of persisting local antigen presentation. Proc Natl Acad Sci U S A (2012) 109(18):7037–42. 10.1073/pnas.1202288109 22509047 PMC3344960

[42] McMaster SR Wilson JJ Wang H Kohlmeier JE Airway-resident memory CD8 T cells provide antigen-specific protection against respiratory virus challenge through rapid IFN-γ production. J Immunol (2015) 195(1):203–9. 10.4049/jimmunol.1402975 26026054 PMC4475417

[43] Mackay LK Minnich M Kragten NAM Liao Y Nota B Seillet C Hobit and Blimp1 instruct a universal transcriptional program of tissue residency in lymphocytes. Science (2016) 352(6284):459–63. 10.1126/science.aad2035 27102484

[44] Milner JJ Toma C Yu B Zhang K Omilusik K Phan AT Runx3 programs CD8+ T cell residency in non-lymphoid tissues and tumours. Nature (2017) 552(7684):253–7. 10.1038/nature24993 29211713 PMC5747964

[45] Li Y Innocentin S Withers David R Roberts Natalie A Gallagher Alec R Grigorieva Elena F Exogenous stimuli maintain intraepithelial lymphocytes via aryl hydrocarbon receptor activation. Cell (2011) 147(3):629–40. 10.1016/j.cell.2011.09.025 21999944

[46] Boddupalli CS Nair S Gray SM Nowyhed HN Verma R Gibson JA ABC transporters and NR4A1 identify a quiescent subset of tissue-resident memory T cells. J Clin Invest (2016) 126(10):3905–16. 10.1172/JCI85329 27617863 PMC5096804

[47] Gerlach C Rohr JC Perie L van Rooij N van Heijst JW Velds A Heterogeneous differentiation patterns of individual CD8+ T cells. Science (2013) 340(6132):635–9. 10.1126/science.1235487 23493421

[48] Stemberger C Huster KM Koffler M Anderl F Schiemann M Wagner H A single naive CD8+ T cell precursor can develop into diverse effector and memory subsets. Immunity (2007) 27(6):985–97. 10.1016/j.immuni.2007.10.012 18082432

[49] Gaide O Emerson RO Jiang X Gulati N Nizza S Desmarais C Common clonal origin of central and resident memory T cells following skin immunization. Nat Med (2015) 21(6):647–53. 10.1038/nm.3860 25962122 PMC4632197

[50] Joshi NS Cui W Chandele A Lee HK Urso DR Hagman J Inflammation directs memory precursor and short-lived effector CD8+ T cell fates via the graded expression of T-bet transcription factor. Immunity (2007) 27(2):281–95. 10.1016/j.immuni.2007.07.010 17723218 PMC2034442

[51] Kaech SM Tan JT Wherry EJ Konieczny BT Surh CD Ahmed R. Selective expression of the interleukin 7 receptor identifies effector CD8 T cells that give rise to long-lived memory cells. Nat Immunol (2003) 4(12):1191–8. 10.1038/ni1009 14625547

[52] Sanjabi S Mosaheb MM Flavell RA. Opposing effects of TGF-beta and IL-15 cytokines control the number of short-lived effector CD8+ T cells. Immunity (2009) 31(1):131–44. 10.1016/j.immuni.2009.04.020 19604492 PMC2765785

[53] Herndler-Brandstetter D Ishigame H Shinnakasu R Plajer V Stecher C Zhao J KLRG1+ effector CD8+ T cells lose KLRG1, differentiate into all memory T cell lineages, and convey enhanced protective immunity. Immunity (2018) 48(4):716–29.e8. 10.1016/j.immuni.2018.03.015 29625895 PMC6465538

[54] Kakaradov B Arsenio J Widjaja CE He Z Aigner S Metz PJ Early transcriptional and epigenetic regulation of CD8+ T cell differentiation revealed by single-cell RNA sequencing. Nat Immunol (2017) 18(4):422–32. 10.1038/ni.3688 28218746 PMC5360497

[55] Masopust D Vezys V Wherry EJ Barber DL Ahmed R. Cutting edge: gut microenvironment promotes differentiation of a unique memory CD8 T cell population. J Immunol (2006) 176(4):2079–83. 10.4049/jimmunol.176.4.2079 16455963

[56] Casey KA Fraser KA Schenkel JM Moran A Abt MC Beura LK Antigen-independent differentiation and maintenance of effector-like resident memory T cells in tissues. J Immunol (2012) 188(10):4866–75. 10.4049/jimmunol.1200402 22504644 PMC3345065

[57] Bergsbaken T Bevan MJ Proinflammatory microenvironments within the intestine regulate the differentiation of tissue-resident CD8+ T cells responding to infection. Nat Immunol (2015) 16:406–14. 10.1038/ni.3108 25706747 PMC4368475

[58] Bergsbaken T Bevan MJ Fink PJ. Local inflammatory cues regulate differentiation and persistence of CD8+ tissue-resident memory T cells. Cell Rep (2017) 19(1):114–24. 10.1016/j.celrep.2017.03.031 28380351 PMC5444811

[59] Iborra S Martinez-Lopez M Khouili SC Enamorado M Cueto FJ Conde-Garrosa R Optimal generation of tissue-resident but not circulating memory T cells during viral infection requires crosspriming by DNGR-1(+) dendritic cells. Immunity (2016) 45(4):847–60. 10.1016/j.immuni.2016.08.019 27692611 PMC5074364

[60] Iijima N Iwasaki A A local macrophage chemokine network sustains protective tissue-resident memory CD4 T cells. Science (2014) 346(6205):93–8. 10.1126/science.1257530 25170048 PMC4254703

[61] Collins N Hochheiser K Carbone FR Gebhardt T. Sustained accumulation of antigen-presenting cells after infection promotes local T-cell immunity. Immunol Cell Biol (2017) 95(10):878–83. 10.1038/icb.2017.60 28722019

[62] McMaster SR Wein AN Dunbar PR Hayward SL Cartwright EK Denning TL Pulmonary antigen encounter regulates the establishment of tissue-resident CD8 memory T cells in the lung airways and parenchyma. Mucosal Immunol (2018) 11(4):1071–8. 10.1038/s41385-018-0003-x 29453412 PMC6030505

[63] Muschaweckh A Buchholz VR Fellenzer A Hessel C König P-A Tao S Antigen-dependent competition shapes the local repertoire of tissue-resident memory CD8+ T cells. J Exp Med (2016) 213:3075–86. 10.1084/jem.20160888 27899444 PMC5154944

[64] Khan TN Mooster JL Kilgore AM Osborn JF Nolz JC Local antigen in nonlymphoid tissue promotes resident memory CD8+ T cell formation during viral infection. J Exp Med (2016) 213:951–66. 10.1084/jem.20151855 27217536 PMC4886364

[65] Davies B Prier JE Jones CM Gebhardt T Carbone FR Mackay LK Cutting edge: tissue-resident memory T cells generated by multiple immunizations or localized deposition provide enhanced immunity. J Immunol (2017) 198(6):2233–7. 10.4049/jimmunol.1601367 28159905

[66] Thom JT Weber TC Walton SM Torti N Oxenius A The salivary gland acts as a sink for tissue-resident memory CD8+ T cells, facilitating protection from local cytomegalovirus infection. Cell Rep (2015) 13(6):1125–36. 10.1016/j.celrep.2015.09.082 26526997

[67] Slütter B Van Braeckel-Budimir N Abboud G Varga SM Salek-Ardakani S Harty JT. Dynamics of influenza-induced lung-resident memory T cells underlie waning heterosubtypic immunity. Sci Immunol (2017) 2(7):eaag2031. 10.1126/sciimmunol.aag2031 28783666 PMC5590757

[68] Zhou AC Wagar LE Wortzman ME Watts TH. Intrinsic 4-1BB signals are indispensable for the establishment of an influenza-specific tissue-resident memory CD8 T-cell population in the lung. Mucosal Immunol (2017) 10(5):1294–309. 10.1038/mi.2016.124 28051085

[69] Liu L Zhong Q Tian T Dubin K Athale SK Kupper TS. Epidermal injury and infection during poxvirus immunization is crucial for the generation of highly protective T cell-mediated immunity. Nat Med (2010) 16(2):224–7. 10.1038/nm.2078 20081864 PMC3070948

[70] Takamura S Yagi H Hakata Y Motozono C McMaster SR Masumoto T Specific niches for lung-resident memory CD8+ T cells at the site of tissue regeneration enable CD69-independent maintenance. J Exp Med (2016) 213(13):3057–73. 10.1084/jem.20160938 27815325 PMC5154946

[71] Takamura S. Niches for the long-term maintenance of tissue-resident memory T cells. Front Immunol (2018) 9(1214):1214. 10.3389/fimmu.2018.01214 29904388 PMC5990602

[72] Zaid A Mackay LK Rahimpour A Braun A Veldhoen M Carbone FR Persistence of skin-resident memory T cells within an epidermal niche. Proc Natl Acad Sci U S A (2014) 111(14):5307–12. 10.1073/pnas.1322292111 24706879 PMC3986170

[73] Wakim LM Gupta N Mintern JD Villadangos JA Enhanced survival of lung tissue-resident memory CD8(+) T cells during infection with influenza virus due to selective expression of IFITM3. Nat Immunol (2013) 14(3):238–45. 10.1038/ni.2525 23354485

[74] Mackay Laura K Wynne-Jones E Freestone D Pellicci Daniel G Mielke Lisa A Newman Dane M T-box transcription factors combine with the cytokines TGF-β and IL-15 to control tissue-resident memory T cell fate. Immunity (2015) 43(6):1101–11. 10.1016/j.immuni.2015.11.008 26682984

[75] Adachi T Kobayashi T Sugihara E Yamada T Ikuta K Pittaluga S Hair follicle–derived IL-7 and IL-15 mediate skin-resident memory T cell homeostasis and lymphoma. Nat Med (2015) 21:1272–9. 10.1038/nm.3962 26479922 PMC4636445

[76] Schenkel JM Fraser KA Casey KA Beura LK Pauken KE Vezys V IL-15-independent maintenance of tissue-resident and boosted effector memory CD8 T cells. J Immunol (2016) 196(9):3920–6. 10.4049/jimmunol.1502337 27001957 PMC5145194

[77] Zhang N Bevan Michael J. Transforming growth factor-β signaling controls the formation and maintenance of gut-resident memory T cells by regulating migration and retention. Immunity (2013) 39(4):687–96. 10.1016/j.immuni.2013.08.019 24076049 PMC3805703

[78] Mohammed J Beura LK Bobr A Astry B Chicoine B Kashem SW Stromal cells control the epithelial residence of DCs and memory T cells by regulated activation of TGF-β. Nat Immunol (2016) 17(4):414–21. 10.1038/ni.3396 26901152 PMC5135085

[79] Wu T Hu Y Lee YT Bouchard KR Benechet A Khanna K Lung-resident memory CD8 T cells (TRM) are indispensable for optimal cross-protection against pulmonary virus infection. J Leukoc Biol (2014) 95(2):215–24. 10.1189/jlb.0313180 24006506 PMC3896663

[80] Zaid A Hor JL Christo SN Groom JR Heath WR Mackay LK Chemokine receptor-dependent control of skin tissue-resident memory T cell formation. J Immunol (2017) 199(7):2451–9. 10.4049/jimmunol.1700571 28855310

[81] Pan Y Tian T Park CO Lofftus SY Mei S Liu X Survival of tissue-resident memory T cells requires exogenous lipid uptake and metabolism. Nature (2017) 543(7644):252–6. 10.1038/nature21379 28219080 PMC5509051

[82] Storch J Thumser AE. Tissue-specific functions in the fatty acid-binding protein family. J Biol Chem (2010) 285(43):32679–83. 10.1074/jbc.R110.135210 20716527 PMC2963392

[83] Gebhardt T Whitney PG Zaid A Mackay LK Brooks AG Heath WR Different patterns of peripheral migration by memory CD4+ and CD8+ T cells. Nature (2011) 477(7363):216–9. 10.1038/nature10339 21841802

[84] Ariotti S Beltman JB Chodaczek G Hoekstra ME van Beek AE Gomez-Eerland R Tissue-resident memory CD8+ T cells continuously patrol skin epithelia to quickly recognize local antigen. Proc Natl Acad Sci U S A (2012) 109(48):19739–44. 10.1073/pnas.1208927109 23150545 PMC3511734

[85] Beura LK Mitchell JS Thompson EA Schenkel JM Mohammed J Wijeyesinghe S Intravital mucosal imaging of CD8+ resident memory T cells shows tissue-autonomous recall responses that amplify secondary memory. Nat Immunol (2018) 19(2):173–82. 10.1038/s41590-017-0029-3 29311694 PMC5896323

[86] Park SL Zaid A Hor JL Christo SN Prier JE Davies B Local proliferation maintains a stable pool of tissue-resident memory T cells after antiviral recall responses. Nat Immunol (2018) 19(2):183–91. 10.1038/s41590-017-0027-5 29311695

[87] Mempel TR Henrickson SE von Andrian UH. T-cell priming by dendritic cells in lymph nodes occurs in three distinct phases. Nature (2004) 427(6970):154–9. 10.1038/nature02238 14712275

[88] Wakim LM Woodward-Davis A Liu R Hu Y Villadangos J Smyth G The molecular signature of tissue resident memory CD8 T cells isolated from the brain. J Immunol (2012) 189(7):3462–71. 10.4049/jimmunol.1201305 22922816 PMC3884813

[89] Anderson P. Post-transcriptional control of cytokine production. Nat Immunol (2008) 9(4):353–9. 10.1038/ni1584 18349815

[90] Salerno F Paolini NA Stark R von Lindern M Wolkers MC. Distinct PKC-mediated posttranscriptional events set cytokine production kinetics in CD8+ T cells. Proc Natl Acad Sci U S A (2017) 114(36):9677–82. 10.1073/pnas.1704227114 28835535 PMC5594653

[91] Schenkel JM Fraser KA Vezys V Masopust D Sensing and alarm function of resident memory CD8+ T cells. Nat Immunol (2013) 14(5):509–13. 10.1038/ni0813-876c 23542740 PMC3631432

[92] Schenkel JM Fraser KA Beura LK Pauken KE Vezys V Masopust D Resident memory CD8 T cells trigger protective innate and adaptive immune responses. Science (2014) 346(6205):98–101. 10.1126/science.1254536 25170049 PMC4449618

[93] Reichel CA Puhr-Westerheide D Zuchtriegel G Uhl B Berberich N Zahler S C-C motif chemokine CCL3 and canonical neutrophil attractants promote neutrophil extravasation through common and distinct mechanisms. Blood (2012) 120(4):880–90. 10.1182/blood-2012-01-402164 22674804

[94] Shi C Pamer EG. Monocyte recruitment during infection and inflammation. Nat Rev Immunol (2011) 11(11):762–74. 10.1038/nri3070 21984070 PMC3947780

[95] Ariotti S Hogenbirk MA Dijkgraaf FE Visser LL Hoekstra ME Song J-Y Skin-resident memory CD8+ T cells trigger a state of tissue-wide pathogen alert. Science (2014) 346(6205):101–5. 10.1126/science.1254803 25278612

[96] Kadoki M Patil A Thaiss CC Brooks DJ Pandey S Deep D Organism-level analysis of vaccination reveals networks of protection across tissues. Cell (2017) 171(2):398–413.e21. 10.1016/j.cell.2017.08.024 28942919 PMC7895295

[97] Steinbach K Vincenti I Kreutzfeldt M Page N Muschaweckh A Wagner I Brain-resident memory T cells represent an autonomous cytotoxic barrier to viral infection. J Exp Med (2016) 213(8):1571–87. 10.1084/jem.20151916 27377586 PMC4986533

[98] Afonina IS Cullen SP Martin SJ. Cytotoxic and non-cytotoxic roles of the CTL/NK protease granzyme B. Immunol Rev (2010) 235(1):105–16. 10.1111/j.0105-2896.2010.00908.x 20536558

[99] Froelich CJ Pardo J Simon MM Granule-associated serine proteases: granzymes might not just be killer proteases. Trends Immunol (2009) 30(3):117–23. 10.1016/j.it.2009.01.002 19217825

[100] Wherry EJ Teichgräber V Becker TC Masopust D Kaech SM Antia R Lineage relationship and protective immunity of memory CD8 T cell subsets. Nat Immunol (2003) 4(3):225–34. 10.1038/ni889 12563257

[101] Roberts AD Ely KH Woodland DL. Differential contributions of central and effector memory T cells to recall responses. J Exp Med (2005) 202(1):123–33. 10.1084/jem.20050137 15983064 PMC2212898

[102] Bouneaud C Garcia Z Kourilsky P Pannetier C Lineage relationships, homeostasis, and recall capacities of central– and effector–memory CD8 T cells in vivo. J Exp Med (2005) 201(4):579–90. 10.1084/jem.20040876 15710650 PMC2213051

[103] Enamorado M Iborra S Priego E Cueto FJ Quintana JA Martínez-Cano S Enhanced anti-tumour immunity requires the interplay between resident and circulating memory CD8+ T cells. Nat Commun (2017) 8:16073. 10.1038/ncomms16073 28714465 PMC5520051

[104] Nolz JC Harty JT. Protective capacity of memory CD8+ T cells is dictated by antigen exposure history and nature of the infection. Immunity (2011) 34(5):781–93. 10.1016/j.immuni.2011.03.020 21549619 PMC3103642

[105] Fraser KA Schenkel JM Jameson SC Vezys V Masopust D. Preexisting high frequencies of memory CD8+ T cells favor rapid memory differentiation and preservation of proliferative potential upon boosting. Immunity (2013) 39(1):171–83. 10.1016/j.immuni.2013.07.003 23890070 PMC3979587

[106] Masopust D Vezys V Usherwood EJ Cauley LS Olson S Marzo AL Activated primary and memory CD8 T cells migrate to nonlymphoid tissues regardless of site of activation or tissue of origin. J Immunol (2004) 172(8):4875–82. 10.4049/jimmunol.172.8.4875 15067066

[107] Pallett LJ Davies J Colbeck EJ Robertson F Hansi N Easom NJW IL-2high tissue-resident T cells in the human liver: sentinels for hepatotropic infection. J Exp Med (2017) 214(6):1567–80. 10.1084/jem.20162115 28526759 PMC5461007

[108] Braga FAV Hertoghs KML Kragten NAM Doody GM Barnes NA Remmerswaal EBM Blimp-1 homolog Hobit identifies effector-type lymphocytes in humans. Eur J Immunol (2015) 45(10):2945–58. 10.1002/eji.201545650 26179882

[109] Bai A Hu H Yeung M Chen J Krüppel-like factor 2 controls T cell trafficking by activating L-selectin (CD62L) and sphingosine-1-phosphate receptor 1 transcription. J Immunol (2007) 178(12):7632–9. 10.4049/jimmunol.178.12.7632 17548599

[110] Matloubian M Lo CG Cinamon G Lesneski MJ Xu Y Brinkmann V Lymphocyte egress from thymus and peripheral lymphoid organs is dependent on S1P receptor 1. Nature (2004) 427(6972):355–60. 10.1038/nature02284 14737169

[111] Takada K Wang X Hart GT Odumade OA Weinreich MA Hogquist KA Kruppel-like factor 2 is required for trafficking but not quiescence in postactivated T cells. J Immunol (2011) 186(2):775–83. 10.4049/jimmunol.1000094 21160050 PMC3017213

[112] Zhou X Yu S Zhao D-M Harty JT Badovinac VP Xue H-H Differentiation and persistence of memory CD8+ T cells depend on T cell factor 1. Immunity (2010) 33(2):229–40. 10.1016/j.immuni.2010.08.002 20727791 PMC2928475

[113] Hertoghs KML Moerland PD van Stijn A Remmerswaal EBM Yong SL van de Berg PJEJ Molecular profiling of cytomegalovirus-induced human CD8+ T cell differentiation. J Clin Invest (2010) 120(11):4077–90. 10.1172/JCI42758 20921622 PMC2964975

[114] Cruz-Guilloty F Pipkin ME Djuretic IM Levanon D Lotem J Lichtenheld MG Runx3 and T-box proteins cooperate to establish the transcriptional program of effector CTLs. J Exp Med (2009) 206(1):51–9. 10.1084/jem.20081242 19139168 PMC2626671

[115] Rutishauser RL Martins GA Kalachikov S Chandele A Parish IA Meffre E Transcriptional repressor Blimp-1 promotes CD8+ T cell terminal differentiation and represses the acquisition of central memory T cell properties. Immunity (2009) 31(2):296–308. 10.1016/j.immuni.2009.05.014 19664941 PMC2783637

[116] Kallies A Xin A Belz GT Nutt SL Blimp-1 transcription factor is required for the differentiation of effector CD8+ T cells and memory responses. Immunity (2009) 31(2):283–95. 10.1016/j.immuni.2009.06.021 19664942

[117] Backer RA Helbig C Gentek R Kent A Laidlaw BJ Dominguez CX A central role for Notch in effector CD8+ T cell differentiation. Nat Immunol (2014) 15(12):1143–51. 10.1038/ni.3027 25344724 PMC4232996

[118] Setoguchi R Tachibana M Naoe Y Muroi S Akiyama K Tezuka C Repression of the transcription factor Th-POK by Runx complexes in cytotoxic T cell development. Science (2008) 319(5864):822–5. 10.1126/science.1151844 18258917

[119] Egawa T Tillman RE Naoe Y Taniuchi I Littman DR. The role of the Runx transcription factors in thymocyte differentiation and in homeostasis of naive T cells. J Exp Med (2007) 204(8):1945–57. 10.1084/jem.20070133 17646406 PMC2118679

[120] Collins A Littman DR Taniuchi I. RUNX proteins in transcription factor networks that regulate T-cell lineage choice. Nat Rev Immunol (2009) 9(2):106–15. 10.1038/nri2489 19165227 PMC4231139

[121] Grueter B Petter M Egawa T Laule-Kilian K Aldrian CJ Wuerch A Runx3 regulates integrin αE/CD103 and CD4 expression during development of CD4−/CD8+ T cells. J Immunol (2005) 175(3):1694–705. 10.4049/jimmunol.175.3.1694 16034110

[122] Wang D Diao H Getzler AJ Rogal W Frederick MA Milner J The transcription factor Runx3 establishes chromatin accessibility of cis-regulatory landscapes that drive memory cytotoxic T lymphocyte formation. Immunity (2018) 48(4):659–74.e6. 10.1016/j.immuni.2018.03.028 29669249 PMC6750808

[123] Backer RA Hombrink P Helbig C Amsen D Chapter 2 – The fate choice between effector and memory T cell lineages: asymmetry, signal integration, and feedback to create bistability. In: Alt F, editor. Advances in Immunology. (Vol. 137), Academic Press (2018). p. 43–82. 10.1016/bs.ai.2017.12.003 29455847

[124] Amsen D Helbig C Backer RA. Notch in T cell differentiation: all things considered. Trends Immunol (2015) 36(12):802–14. 10.1016/j.it.2015.10.007 26617322

[125] Elyaman W Bassil R Bradshaw Elizabeth M Orent W Lahoud Y Zhu B Notch receptors and Smad3 signaling cooperate in the induction of interleukin-9-producing T cells. Immunity (2012) 36(4):623–34. 10.1016/j.immuni.2012.01.020 22503540 PMC3572366

[126] Blokzijl A Dahlqvist C Reissmann E Falk A Moliner A Lendahl U Cross-talk between the Notch and TGF-β signaling pathways mediated by interaction of the Notch intracellular domain with Smad3. J Cell Biol (2003) 163(4):723–8. 10.1083/jcb.200305112 14638857 PMC2173673

[127] Intlekofer AM Takemoto N Wherry EJ Longworth SA Northrup JT Palanivel VR Effector and memory CD8+ T cell fate coupled by T-bet and eomesodermin. Nat Immunol (2005) 6(12):1236–44. 10.1038/ni1268 16273099

[128] Laidlaw Brian J Zhang N Marshall Heather D Staron Mathew M Guan T Hu Y CD4+ T cell help guides formation of CD103+ lung-resident memory CD8+ T cells during influenza viral infection. Immunity (2014) 41(4):633–45. 10.1016/j.immuni.2014.09.007 25308332 PMC4324721

[129] Shan Q Zeng Z Xing S Li F Hartwig SM Gullicksrud JA The transcription factor Runx3 guards cytotoxic CD8+ effector T cells against deviation towards follicular helper T cell lineage. Nat Immunol (2017) 18(8):931–9. 10.1038/ni.3773 28604718 PMC5564218

[130] van Gisbergen KPJM Kragten NAM Hertoghs KML Wensveen FM Jonjic S Hamann J Mouse Hobit is a homolog of the transcriptional repressor Blimp-1 that regulates NKT cell effector differentiation. Nat Immunol (2012) 13:864. 10.1038/ni.2393 22885984

[131] Pearce EL Mullen AC Martins GA Krawczyk CM Hutchins AS Zediak VP Control of effector CD8+ T cell function by the transcription factor eomesodermin. Science (2003) 302(5647):1041–3. 10.1126/science.1090148 14605368

[132] Kohlmeier JE Cookenham T Roberts AD Miller SC Woodland DL. Type I interferons regulate cytolytic activity of memory CD8(+) T cells in the lung airways during respiratory virus challenge. Immunity (2010) 33(1):96–105. 10.1016/j.immuni.2010.06.016 20637658 PMC2908370

[133] Joshi NS Cui W Dominguez CX Chen JH Hand TW Kaech SM. Increased numbers of preexisting memory CD8 T cells and decreased T-bet expression can restrain terminal differentiation of secondary effector and memory CD8 T cells. J Immunol (2011) 187(8):4068–76. 10.4049/jimmunol.1002145 21930973 PMC3991478

[134] Xin A Masson F Liao Y Preston S Guan T Gloury R A molecular threshold for effector CD8+ T cell differentiation controlled by transcription factors Blimp-1 and T-bet. Nat Immunol (2016) 17:422–32. 10.1038/ni.3410 26950239 PMC5779087

[135] Prigent L Robineau M Jouneau S Morzadec C Louarn L Vernhet L The aryl hydrocarbon receptor is functionally upregulated early in the course of human T-cell activation. Eur J Immunol (2014) 44(5):1330–40. 10.1002/eji.201343920 24549985

[136] Au-Yeung BB Smith GA Mueller JL Heyn CS Jaszczak RG Weiss A IL-2 Modulates the TCR signaling threshold for CD8 but not CD4 T cell proliferation on a single-cell level. J Immunol (2017) 198(6):2445–56. 10.4049/jimmunol.1601453 28159902 PMC5340617

[137] Nowyhed HN Huynh TR Thomas GD Blatchley A Hedrick CC. Cutting edge: the orphan nuclear receptor Nr4a1 regulates CD8+ T cell expansion and effector function through direct repression of Irf4. J Immunol (2015) 195(8):3515–9. 10.4049/jimmunol.1403027 26363057 PMC4592102

[138] Çuburu N Graham BS Buck CB Kines RC Pang Y-YS Day PM Intravaginal immunization with HPV vectors induces tissue-resident CD8+ T cell responses. J Clin Invest (2012) 122(12):4606–20. 10.1172/JCI63287 23143305 PMC3533540

[139] Nizard M Roussel H Diniz MO Karaki S Tran T Voron T Induction of resident memory T cells enhances the efficacy of cancer vaccine. Nat Commun (2017) 8:15221. 10.1038/ncomms15221 28537262 PMC5458068

[140] Zens KD Chen JK Farber DL Vaccine-generated lung tissue-resident memory T cells provide heterosubtypic protection to influenza infection. JCI Insight (2016) 1(10):e85832 10.1172/jci.insight.85832 27468427 PMC4959801

[141] Sun Y-Y Peng S Han L Qiu J Song L Tsai Y Local HPV recombinant vaccinia boost following priming with an HPV DNA vaccine enhances local HPV-specific CD8+ T-cell-mediated tumor control in the genital tract. Clin Cancer Res (2016) 22(3):657–69. 10.1158/1078-0432.CCR-15-0234 26420854 PMC4738102

[142] Malik BT Byrne KT Vella JL Zhang P Shabaneh TB Steinberg SM Resident memory T cells in the skin mediate durable immunity to melanoma. Sci Immunol (2017) 2(10):eaam6346. 10.1126/sciimmunol.aam6346 28738020 PMC5525335

[143] Djenidi F Adam J Goubar A Durgeau A Meurice G de Montpréville V CD8+ CD103+ tumor-infiltrating lymphocytes are tumor-specific tissue-resident memory T cells and a prognostic factor for survival in lung cancer patients. J Immunol (2015) 194(7):3475–86. 10.4049/jimmunol.1402711 25725111

[144] Ganesan A-P Clarke J Wood O Garrido-Martin EM Chee SJ Mellows T Tissue-resident memory features are linked to the magnitude of cytotoxic T cell responses in human lung cancer. Nat Immunol (2017) 18(8):940–50. 10.1038/ni.3775 28628092 PMC6036910

[145] Koh J Kim S Kim M-Y Go H Jeon YK Chung DH Prognostic implications of intratumoral CD103(+) tumor-infiltrating lymphocytes in pulmonary squamous cell carcinoma. Oncotarget (2017) 8(8):13762–9. 10.18632/oncotarget.14632 28099920 PMC5355136

